# Influence of Desialylation on the Drug Binding Affinity of Human Alpha-1-Acid Glycoprotein Assessed by Microscale Thermophoresis

**DOI:** 10.3390/pharmaceutics16020230

**Published:** 2024-02-05

**Authors:** Tino Šeba, Robert Kerep, Tin Weitner, Dinko Šoić, Toma Keser, Gordan Lauc, Mario Gabričević

**Affiliations:** 1 Department of General and Inorganic Chemistry, Faculty of Pharmacy and Biochemistry, University of Zagreb, A. Kovačića 1, 10000 Zagreb, Croatia; tseba@pharma.hr (T.Š.); rkerep@pharma.hr (R.K.); tweitner@pharma.hr (T.W.); 2 Department of Biochemistry and Molecular Biology, Faculty of Pharmacy and Biochemistry, University of Zagreb, A. Kovačića 1, 10000 Zagreb, Croatia; dsoic@pharma.hr (D.Š.); toma.keser@pharma.unizg.hr (T.K.); glauc@pharma.hr (G.L.)

**Keywords:** alpha-1-acid glycoprotein, plasma protein binding, equilibrium binding affinity, desialylation, microscale thermophoresis

## Abstract

Human serum alpha-1-acid glycoprotein (AAG) is an acute-phase plasma protein involved in the binding and transport of many drugs, especially basic and lipophilic substances. The sialic acid groups that terminate the N-glycan chains of AAG have been reported to change in response to numerous health conditions and may have an impact on the binding of drugs to AAG. In this study, we quantified the binding between native and desialylated AAG and seven drugs from different pharmacotherapeutic groups (carvedilol, diltiazem, dipyridamole, imipramine, lidocaine, propranolol, vinblastine) using microscale thermophoresis (MST). This method was chosen due to its robustness and high sensitivity, allowing precise quantification of molecular interactions based on the thermophoretic movement of fluorescent molecules. Detailed glycan analysis of native and desialylated AAG showed over 98% reduction in sialic acid content for the enzymatically desialylated AAG. The MST results indicate that desialylation generally alters the binding affinity between AAG and drugs, leading to either an increase or decrease in *K*_d_ values, probably due to conformational changes of AAG caused by the different sialic acid content. This effect is also reflected in an increased denaturation temperature of desialylated AAG. Our findings indicate that desialylation impacts free drug concentrations differently, depending on the binding affinity of the drug with AAG relative to human serum albumin (HSA). For drugs such as dipyridamole, lidocaine, and carvedilol, which have a higher affinity for AAG, desialylation significantly changes free drug concentrations. In contrast, drugs such as propranolol, imipramine, and vinblastine, which have a strong albumin binding, show only minimal changes. It is noteworthy that the free drug concentration of dipyridamole is particularly sensitive to changes in AAG concentration and glycosylation, with a decrease of up to 15% being observed, underscoring the need for dosage adjustments in personalized medicine.

## 1. Introduction

The binding of drugs to plasma proteins plays a significant role in drug pharmacokinetics and pharmacodynamics, as it can affect drug distribution, metabolism, and excretion, and ultimately its therapeutic effect and toxicity [1,2]. The ‘free drug hypothesis’ states that only the unbound or ‘free’ fraction of a drug can interact with its molecular target and produce a therapeutic effect. On the contrary, the bound fraction is inactive and acts as a reservoir that can be released to maintain a constant concentration of free drug [3]. Therefore, a comprehensive understanding of the interactions between drugs and plasma proteins is essential for the optimization of therapy.

Human alpha-1-acid glycoprotein (AAG) or orosomucoid (ORM) is a single-chain highly glycosylated plasma protein synthesized primarily in the liver. It belongs to a group of alpha-1-globulins, together with alpha-1-antitrypsin and alpha-1-fetoglobulin [4]. It consists of 183 amino acid residues with two cystine bridges and up to five N-glycans bound to asparagine residues [5]. Along with human serum albumin (HSA), AAG is the major contributor to plasma protein binding. However, in the past, the role of AAG in plasma protein binding has been underestimated due to its lower plasma concentration (0.36–1.46 g/L) compared to HSA (35–50 g/L) [6,7,8]. It has been found that its concentration in plasma is sex-dependent, increases with age, and during various pathological conditions, such as inflammation, infection, and cancer [5,7,9,10]. Therefore, it is considered one of the major acute-phase proteins. Although the physiological role of AAG is not fully understood, it is believed to play a role in both pro- and anti-inflammatory responses [5,11,12]. AAG is also responsible for binding various basic and neutral lipophilic endobiotics and xenobiotics, further highlighting the importance of characterizing AAG–drug interactions to improve drug efficacy and safety [13].

The expression of two main AAG variants, ORM1 and ORM2, is regulated by three genes on chromosome 9 (AAG-A, AAG-B, and AAG-B’). AAG-A encodes polymorphic ORM1, which includes three closely related genetic variants (F1, F2, and S), differing by less than five amino acids in the polypeptide structure. Genetic variants A (ORM2) are encoded by the AAG-B and AAG-B’ genes, which differ from ORM1 by 22 amino acids [13]. These structural differences between AAG genetic variants can affect drug binding affinity and stereoselectivity [14,15,16]. Individuals typically exhibit a combination of AAG genetic variants. The predominant phenotype, found in about 50% of individuals, is a combination of F1, S, and A variants. This is followed by the F1 + A phenotype, present in approximately 35% of individuals, and the S + A phenotype, observed in about 15%. The relative proportion of ORM1 to ORM2 variants in the bloodstream generally ranges from approximately 2:1 to 3:1. However, this ratio can shift significantly in response to certain diseases, with the potential to increase to as much as 8:1, primarily due to the inducible nature of ORM1 [15].

Glycan microheterogeneity is an additional factor that affects drug binding affinity [5,14]. The AAG glycan content accounts for nearly 45% of its molecular weight. Glycans in plasma are predominantly N-glycans, as they are linked via an amide bond to the protein asparagine sidechain, which is a part of the AsN-X-Ser/Thr/Cys sequence, where X can be any amino acid except Pro [15]. Glycans bound to human AAG are bi-, tri-, and tetra-antenary, with varying degree of fucosylation and sialylation [16,17]. Sialic acid, in the form of N-acetylneuraminic acid, typically terminates N-glycan branches and constitutes up to 10–12% of the total monosaccharides. The high sialic content gives AAG a low isoelectric point [5], contributes to its negative surface charge, and affects the nature of interactions with biological membranes [18]. 

In addition to changes in concentration, various physiological and pathophysiological conditions such as diabetes, asthma, liver disease, lung cancer, severe rheumatoid arthritis, and pregnancy have been shown to affect AAG glycan content [19,20,21,22,23,24,25]. Although some research suggests that desialylation of AAG may affect the affinity and stereoselectivity of drug binding [26,27,28,29,30,31,32,33], further studies are needed to draw a definitive conclusion on the effects of altered AAG glycosylation on drug binding affinity. Since it is well-known that protein binding influences both pharmacokinetic and pharmacodynamic properties of drugs and affects drug absorption, distribution, metabolism, and elimination [1,2], clinical significance of changes in the sialylation of AAG in general should not be neglected.

This is particularly critical for drugs with narrow therapeutic windows, where small changes in the concentration of the free drug can lead to either subtherapeutic effects or toxicity. The binding affinity of AAG to different drugs, which can be altered by changes in sialylation, can have a significant impact on the choice of the most appropriate drug modality for a given patient, particularly regarding drug type, dosing regimen, and personalized medicine approaches. In the context of personalized medicine, assessing the sialylation status of AAG can provide valuable insights for tailoring drug therapy to individual patients. This approach could be particularly beneficial for patients with chronic inflammatory diseases, cancer, or other conditions that significantly alter AAG levels and sialylation patterns. By integrating knowledge of AAG sialylation status into pharmacokinetic models, clinicians can make more informed drug dosing decisions, ultimately improving patient care and outcomes.

This study employs microscale thermophoresis (MST) for quantification of the protein–small molecule binding affinities. MST offers several advantages over commonly used non-fluorescence methods for binding characterization, such as isothermal titration calorimetry or surface plasmon resonance, in terms of low sample consumption and an immobilization-free approach, respectively. In the MST assay, the change in fluorescence is recorded as a function of temperature and ligand concentration. The fluorescence change in response to heat is the result of two effects, temperature-related intensity change (TRIC) and thermophoresis. Fluorophores exhibit temperature-dependent fluorescence variation depending on their chemical environment. Thus, binding events that alter their conformation or physicochemical properties can be directly quantified based on TRIC [34]. Additionally, molecules display directed movement through a spatial temperature gradient, called thermophoresis, which depends on the particle size, the square of the effective surface charge, and hydration entropy. All three parameters can be affected by the binding event [35]. Both TRIC and thermophoresis contribute to the measured MST signal during temperature changes, but their differentiation is irrelevant when interaction parameters are concerned.

According to the available literature, this study is one of the first studies to investigate the impact of AAG desialylation on the equilibrium binding affinity of drugs from various therapeutic groups. The equilibrium binding constants between native and desialylated AAG with carvedilol, diltiazem, dipyridamole, imipramine, lidocaine, propranolol, and vinblastine were determined by MST. 

## 2. Materials and Methods

### 2.1. Materials

Native human AAG (lot no. SLBJ6840V), carvedilol, (+)-*cis*-diltiazem hydrochloride, dipyridamole, vinblastine sulfate, (±)-propranolol hydrochloride, imipramine hydrochloride, lidocaine, and DMSO were purchased from Sigma-Aldrich (St. Louis, MO, USA). All standards had a specified purity of ≥97% and have been used without further purification. Sodium phosphate dihydrate and sodium hydroxide were purchased from Kemika (Zagreb, Croatia). Tween^®^ 20 was purchased from Fischer Scientific (Pittsburgh, PA, USA). Guanidine hydrochloride was purchased from AppliChem GmbH (Darmstadt, Germany). Ultrapure water was obtained using SG Ultra Clear UV Plus (SG, Burladingen, Germany). All other chemicals used in the study were of analytical grade or higher purity. 

### 2.2. Enzymatic Desialylation of Native Human AAG

Desialylated human AAG was prepared by incubation of native human AAG in an Immobilized SialEXO^®^ Microspin column (Genovis AB, Kävlinge, Sweden). Briefly, the column was equilibrated three times with 300 µL of reaction buffer (20 mM sodium phosphate buffer; pH 6.8) and centrifuged at 200 *g* for 1 min. Approximately 0.5 mg of native human AAG dissolved in 300 µL of reaction buffer was added to the column and incubated for 30 min at room temperature with end-over-end mixing. Desialylated AAG was collected in a collection tube using a centrifuge at 1000× *g* for 1 min. Detailed instructions can be found on the manufacturer’s website [36].

Complete glycan profiles of native and desialylated AAG samples were determined using UPLC N-glycan analysis. Briefly, released and fluorescently labeled N-glycans were separated by hydrophilic interaction chromatography on an Acquity UPLC H-Class instrument (Waters, Milford, MA, USA) consisting of a quaternary solvent manager, sample manager, and a fluorescence detector set with excitation and emission wavelengths of 250 and 428 nm, respectively. The instrument was under the control of Empower 3 software, build 3471 (Waters, Milford, MA, USA). Labeled N-glycans were separated on a Waters BEH Glycan chromatography column, with 100 mM ammonium formate, pH 4.4, as solvent A and ACN as solvent B. The separation method used a linear gradient of 70–53% acetonitrile at a flow rate of 0.56 mL/min in a 25 min analytical run. Full details of the protein characterization by UPLC-MS have been described elsewhere [37]. The results were assigned based on N-glycan retention times previously obtained by UPLC-MS/MS analysis [38]. The assigned peaks were integrated using a custom Python script. The np.trapz() function from the NumPy library was used for the integration, which allows integration according to the composite trapezoidal rule [39].

### 2.3. UV–VIS Characterization of Native and Desialylated AAG

To accurately determine AAG concentration, the molar absorption coefficients of native and desialylated AAG were determined using the Edelhoch method, which was described by Gill and von Hippel based on the original data from Edelhoch [40,41]. Briefly, by measuring AAG absorbance at 280 nm ${(A}_{f\;280})$ in 25 mM sodium phosphate buffer (pH 7.4) and AAG absorbance at 280 nm ${(A}_{u\;280})$ dissolved in the same buffer containing denaturation agent–6 M guanidine hydrochloride, the molar absorption coefficient at 280 nm $\left(\varepsilon_{f\;280}\right)$ was calculated as a product of the unfolded molar absorption coefficient ${(\varepsilon }_{u\;280})$ and the absorbance ratio of folded and unfolded AAG $\left(\frac{A_{f\;280}}{A_{u\;280}}\right)$, i.e., Equation (1):

(1) $$\varepsilon_{f\;280}=\varepsilon_{u\;280}\cdot \frac{A_{f\;280}}{A_{u\;280}}$$

The molar absorption coefficient of unfolded AAG $\left(\varepsilon_{u\;280}\right)$ can be calculated from the contribution of four tryptophane, eleven tyrosine, and two cystine residues in the AAG structure [42] using Equation (2):

(2) $$\varepsilon_{u\;280}=N_{Trp}\varepsilon_{Trp\;280}+N_{Tyr}\varepsilon_{Tyr\;280}+N_{Cys}\varepsilon_{Cys\;280}$$

where *N*_Trp_, *N*_Tyr_, and *N*_Cys_ are the number of tryptophane, tyrosine, and cystine residues in AAG, respectively; $\varepsilon_{Trp\;280}$, $\varepsilon_{Tyr\;280}$, and $\varepsilon_{Cys\;280}$ are molar absorption coefficients of tryptophane, tyrosine, and cystine model compounds in 6 M guanidine hydrochloride at pH 6.5. A detailed protocol is described elsewhere [43].

UV/Vis measurements were made on a Varian Carry 50 spectrophotometer (Varian, Palo Alto, CA, USA) using a quartz cuvette with path length *d* = 1 cm (Hellma, Müllheim, Germany). All samples were prepared in triplicates.

### 2.4. AAG Labeling Procedure

Native and desialylated AAG were labeled using the RED-NHS 2nd Generation protein labeling kit (NanoTemper Technologies GmbH, Munich, Germany). The labeling reaction was performed according to the manufacturer’s instructions in the supplied labeling buffer (130 mM NaHCO_3_, 50 mM NaCl, pH 8.2–8.3 at room temperature) by applying 90 µL of 10 µM AAG (molar dye:AAG = 3:1) at room temperature for 30 min. The labeled protein was purified using the B-column provided by the labeling kit. The AAG concentration *c*_AAG_ and the degree of labeling (*DOL*) were calculated using Equations (3) and (4):

(3) $$c_{AAG}\left(M\right)=\frac{A_{280}-(A_{650}\times 0.04)}{\varepsilon_{AGP\;280}\times d}$$

(4) $$DOL=\frac{A_{650}}{195\times 10^{3}M^{-1}{cm}^{-1}\times c_{AAG}\left(M\right)}$$

where $\varepsilon_{AAG\;280}$ is the AAG molar absorption coefficient at 280 nm, *d* is path length of a spectrophotometer in cm, and 0.04 is a correction factor at 280 nm. Aliquoted and flash-frozen labeled protein was stored in liquid nitrogen.

### 2.5. Microscale Thermophoresis Binding Assay

Drug serial dilution of a minimum of 12 data points was prepared using 25 mM sodium phosphate buffer, pH 7.4, supplemented with 0.05% Tween^®^ 20 and 10% DMSO. Each drug dilution was mixed with an equal volume of labeled AAG dissolved in 25 mM sodium phosphate buffer supplemented with 0.05% Tween^®^ 20, leading to a final AAG concentration of 20 nM and a final DMSO content of 5%.

The samples were incubated for 15 min and loaded into Monolith NT.Automated Capillary Chips (NanoTemper Technologies GmbH, Munich, Germany). The MST was measured using a Monolith NT.Automated instrument (NanoTemper Technologies GmbH, Munich, Germany) at room temperature. Instrument parameters were adjusted to 40% LED power and medium MST power. Data of at least two independently pipetted measurements were analyzed with MO.Affinity Analysis v2.3 (NanoTemper Technologies GmbH, Munich, Germany) using the signal from MST on time of 5 s. 

### 2.6. Thermal Shift Assay

Freshly prepared solutions of native and desialylated AAG (1 mg/mL) in 25 mM sodium phosphate buffer, pH 7.4, were loaded into NT.6 capillaries (NanoTemper Technologies GmbH, Munich, Germany). Thermal shift assay was performed using Tycho NT.6 (NanoTemper Technologies GmbH, Munich, Germany) with a constant heating rate of 30 °C /min from 35 °C to 95 °C. The fluorescence signal (ratio 350 nm/330 nm) was recorded as a function of temperature and the resulting curve was automatically analyzed for inflection temperature (*T*_i_).

### 2.7. Statistical Analysis

All results are expressed as means of at least two replicate measurements. Uncertainties are reported as standard deviations. The Student’s t-distribution is used in the hypothesis testing. All *p*-values were calculated using the T.DIST.2T function in Microsoft Excel Version 2401 (Microsoft Corporation, Washington, DC, USA). A small *p*-value (*p* < 0.05) suggests strong evidence against the null hypothesis, whereas a larger *p*-value (0.05 < *p* < 0.1) indicates weaker evidence against the null hypothesis. 

## 3. Results and Discussion

### 3.1. Enzymatic Desialylation and Glycan Analysis of Native and Desialylated Human AAG

The original desialylation protocol was modified to avoid potential buffer incompatibility during the labeling procedure. This was carried out by replacing TRIS with 20 mM sodium phosphate at pH 6.8 as the digestion buffer. The aim was to minimize interference caused by the reactive NHS ester group present in the RED-NHS second protein labeling kit, which binds covalently to primary amines like TRIS.

**Figure 1:**
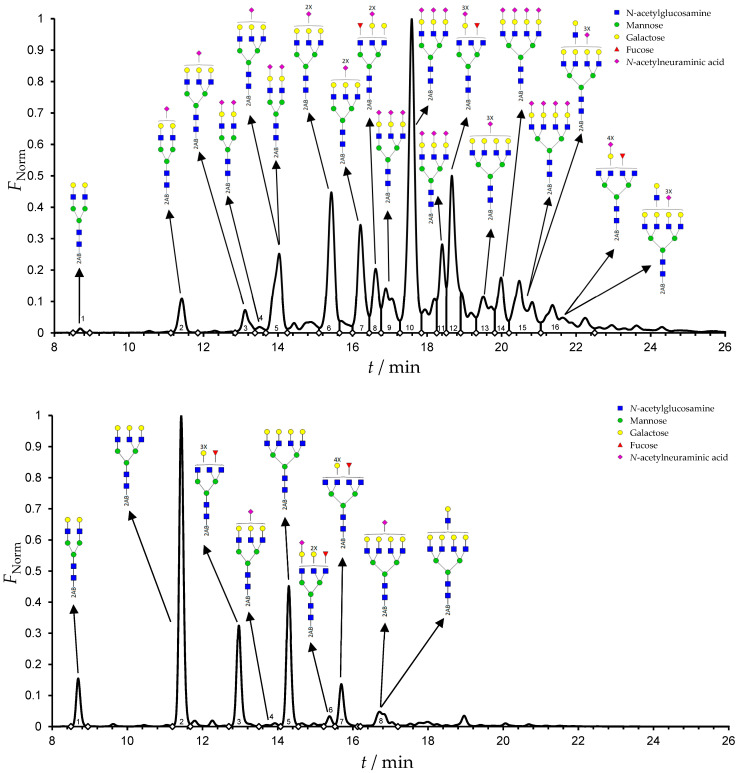
Normalized UPLC chromatogram of fluorescently labeled and purified N-glycans. Fluorescence was recorded using an excitation wavelength of 250 nm and an emission wavelength of 482 nm. The numbers in the figure correspond to the Peak No. in Table 1. The top panel represents native human AAG sample; bottom panel represents desialylated human AAG sample.

Results from the UPLC N-glycan analysis of native and desialylated AAG are presented in Figure 1 and Table 1. The assigned peaks in the chromatograms account for more than 87% of the total integral for native AAG and over 88% for desialylated AAG. Table 1 provides an overview of the relative abundance of AAG N-glycan structures, revealing that tri-antennary glycans are the most abundant, followed by tetra-antennary and di-antennary glycans. Within these structures, the glycans with the highest sialic acid content are the most frequently observed. Conversely, as the number of sialic acids decreases, the occurrence of such glycans decreases accordingly.

To quantify sialic acid content in native and desialylated AAG, the index of sialylation (*IS*) was used. The *IS* value was calculated using Equation (5):

(5) $$IS=\sum_{i=1}^{n}f_{i}\cdot s_{i}$$

where *n* represents the number of N-glycan fractions, *f*_i_ is the percentage of a specific N-glycan fraction of the total assigned N-glycans, and *s*_i_ is the number of sialic acids present in the structure of each N-glycan fraction [44].

**Table 1:** N-glycan composition and content in the native and desialylated human AAG.

Native AAG *IS* = 270			
Peak No.	Content/%	N-glycan Composition *	Schematic N-glycan Structure **
1	0.23	A2G2	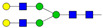
2	2.23	A2G2S1	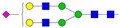
3	1.71	A3G3S1	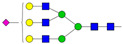
4	0.45	A2G2S2	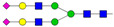
5	6.79	A2G2S2	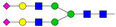
		A3G3S1	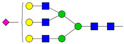
6	10.08	A3G3S2	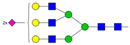
7	7.68	A3G3S2	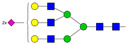
8	4.35	A3F1G3S2	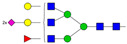
9	5.09	A3G3S3	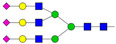
10	21.84	A3G3S3	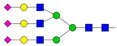
11	5.33	A3G3S3	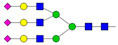
12	11.47	A3F1G3S3	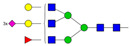
13	4.44	A4G4S3	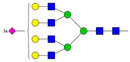
14	4.17	A4G4S4	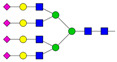
15	7.71	A4G4S4	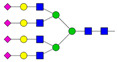
		AFG4S3Lac	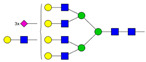
16	6.41	AFG4S3Lac	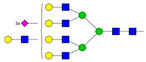
		A4F1G4S4	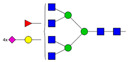
**Desialylated AAG** ***IS* = 5**			
**Peak No.**	**Content/%**	**N-glycan** Composition *	**Schematic** N-glycan Structure **
1	6.48	A2G2	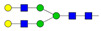
2	43.03	A3G3	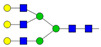
3	15.41	A3F1G3	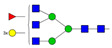
4	0.88	A3G3S1	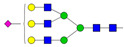
5	20.46	A4G4	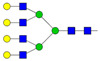
6	1.67	A3F1G3S1	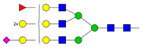
7	6.89	A4F1G4	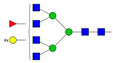
8	5.18	A4G4S1	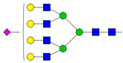
		A4G4Lac	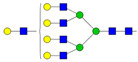

* Structure abbreviations: all N-glycans have two core GlcNAcs; Ax, number of antenna (GlcNAc) on trimannosyl core; A2, biantennary with both GlcNAcs as β1,2-linked; A3, triantennary with a GlcNAc-linked β1,2 to both mannose and the third GlcNAc-linked β1,4 to the α1,3-linked mannose; A4, GlcNAcs linked as A3 with additional GlcNAc β1,6 linked to α1,6 mannose; Gx, number (x) of β1,4-linked galactose on antenna; F(x), number (x) of fucose-linked α1,3 to antenna GlcNAc; Sx, number (x) of sialic acids linked to galactose; Lac(x), number (x) of lactosamine (Galβ1-4GlcNAc) extensions [45]; ** Schematic N-glycan structures: N-acetylglucosamine (■), mannose (●), galactose (●), fucose (▶), sialic acid (◆).

The calculated *IS* values for native and desialylated AAG samples are approximately 270 and 5, respectively, indicating over 98% reduction in the sialic acid content for the enzymatically desialylated AAG. Alternatively, the average number of sialic acids per glycan structure (${\bar{n}}_{glycan}$) can be calculated using the following Equation (6):

(6) $${\bar{n}}_{glycan}=\sum_{i=1}^{n}\frac{f_{i}}{100}\cdot s_{i}$$

AAG has five N-glycan binding sites (*n_s_*_ites_), and the average number of sialic acids on AAG (${\bar{n}}_{AAG}$) was calculated using Equation (7):

(7) $${\bar{n}}_{native\;AAG}={\bar{n}}_{glycan}\cdot n_{sites}=13.5$$

### 3.2. Molar Absorption Coefficient of Native and Desialylated AAG

To the best of our knowledge, molar absorption coefficients of native and desialylated AAG using the Edelhoch method have not been previously reported. It is worth noting that commercially obtained protein samples may contain water; hence, weighing proteins is not an appropriate method for determining protein concentration. Table 2 presents the molar absorption coefficients for both native and desialylated AAG.

The obtained *p*-value of 0.97 indicates that there is no statistically significant difference in the molar absorption coefficients between native and desialylated AAG at 280 nm.

In contrast to the previously described protocol for measurement of the molar absorption coefficients [43], we opted to use the same buffer as in MST experiments (25 mM sodium phosphate buffer at pH 7.4) instead of the recommended 20 mM sodium phosphate buffer at pH 6.5. The change in buffer pH is not expected to impact the determination of molar absorption coefficients because the Trp and Cys side chains do not ionize, and the Tyr side chain has a p*K*_a_ of 10.07 [46]. At pH 7.4, approximately 99.8% of the Tyr side chains are not ionized, making it suitable for the determination of the protein molar absorption coefficients. Moreover, the use of a 25 mM sodium phosphate buffer instead of a 20 mM buffer is not anticipated to affect the molar absorption coefficient of a protein. In our previous study, we demonstrated that the absorbance of native human transferrin exhibits no significant dependence on changes in ionic strength up to 1.5 M [44].

It is important to note that our results are consistent with the reported molar absorption coefficient by K. Schmid [47]. Additionally, they align well with the molar absorption coefficient of folded AAG at 280 nm, which was calculated using the equation derived from the molar absorption coefficients of 80 proteins proposed by Pace et al. [48] (Equation (8)):

(8) $$\frac{\varepsilon_{f\;280}}{M^{-1}{cm}^{-1}}\left(AAG\right)=N_{Trp}\cdot 5500+N_{Tyr}1490+N_{Cys}125=38,640$$

Although the difference between measured and predicted molar absorption coefficients is only around 1%, it is still recommended to experimentally determine $\varepsilon_{280}$, as previous studies have indicated a potential discrepancy of up to 18% between measured and predicted values, particularly for proteins deficient in Trp residues [48].

### 3.3. Desialylation Effect on AAG–Drug Binding Affinity Using MST

To assess the influence of desialylation on AAG–drug binding affinity using MST, we initially labeled both native and desialylated AAG and determined *DOL* using Equation (4). The calculated *DOL* values were approximately 0.45 and 0.77 for native and desialylated AAG, respectively. These results are consistent with the recommended *DOL* range of 0.5 to 1, as suggested by the manufacturer. It is important to avoid *DOL* values below 0.5, as they can result in a lower signal-to-noise ratio, while *DOL* values above 1 may adversely affect protein function. To address the borderline *DOL* of native AAG, a 40% LED power was used to ensure an adequate fluorescence signal without causing a decay of fluorescence intensity due to excitation light-induced photobleaching.

**Figure 2:**
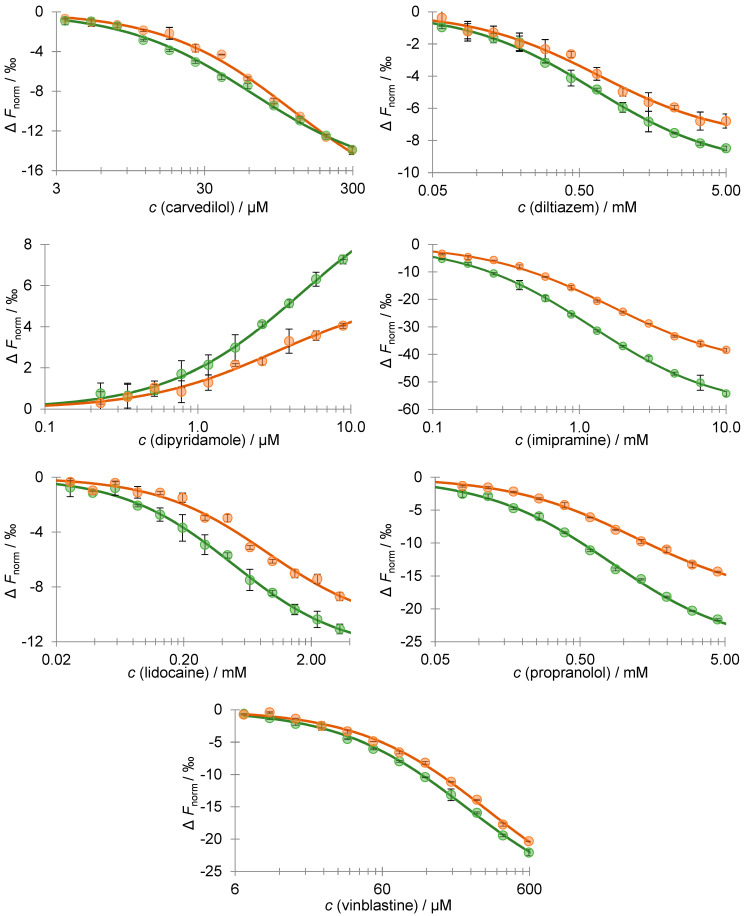
The interaction of drugs with native (green dots) and desialylated AAG (orange dots) was assessed using MST. A titration series of drugs was performed while the labeled AAG was kept constant (20 nM). The solid line represents the theoretical fit of the data. Error bars indicate the standard deviation for each data point, which was calculated based on two independent measurements.

Data obtained in the MST binding assay, as shown in Figure 2, are analyzed using MO.Affinity Analysis software v2.3, which offers a *K*_d_ fit model which describes a molecular interaction with 1:1 stoichiometry according to the law of mass action. The *K*_d_ is estimated by fitting Equation (9):

(9) $$f\left(c\right)=unbound+\left(\;bound-unbound\;\right)\times \frac{\sqrt{{\left(c+c_{target}+K_{d}\right)}^{2}-4\;c\;c_{target}}}{2\;c_{target}}$$

where *f*(*c*) is the fraction bound at the given ligand concentration *c*, *unbound* is the *F*_norm_ signal of the target alone, *bound* is the *F*_norm_ signal of the complex, *K*_d_ is the equilibrium binding affinity, and *c_target_* is the final concentration of the target in the assay. The results are summarized in Table 3.

**Figure 3:**
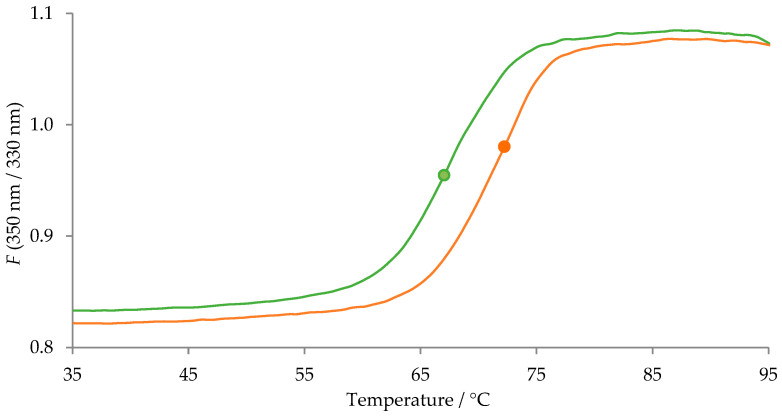
The recorded fluorescence signal of native (green line) and desialylated (orange line) AAG (1 mg/mL) as a function of temperature. The measurements were taken in 25 mM sodium phosphate buffer at pH 7.4. Inflection temperatures of native (green dot) and desialylated (orange dot) AAG are 67.0 and 72.2 °C, respectively.

The results revealed that, in most cases, the desialylated AAG–drug equilibrium binding constants are significantly different (*p* < 0.1) compared to their native counterparts. The obtained MST equilibrium AAG–drug binding curves showed that desialylation of AAG resulted in an increased *K*_d_ for imipramine, lidocaine, propranolol, and vinblastine, a decreased *K*_d_ for carvedilol, and no significant effect on the *K*_d_ for diltiazem. It is important to note that only a few papers addressing the effect of desialylation on AAG–drug equilibrium binding constants have been published to date [26,27,28,29,30,31,32,33]. In general, our results are consistent with previously reported binding studies. Most of these studies showed a slight effect of desialylation on the binding of certain drugs, while others remained unaffected. We also observed a slight decrease in the binding affinity for propranolol upon desialylation, which is consistent with several other studies. However, it should be noted that our experiment yielded lower *K*_d_ values compared to previously reported values, which may be attributed to the use of a more sensitive method [27,29,32]. Furthermore, a previous study has shown that desialylation induces conformational changes in two AAG regions located outside of the ligand-binding pocket (residues 9–18 and 80–89) [49]. From the thermal shift assay depicted in Figure 3, a significant thermal stabilization of desialylated AAG can be observed.

### 3.4. Effect of AAG Desialylation on the Free Drug Fraction

As mentioned above, there are fluctuations in AAG plasma concentration and sialylation during the acute phase, both of which may affect the concentration of free drugs. In addition, the drugs normally bind to both AAG and human serum albumin (HSA). Therefore, both proteins were included in the simulation of the free drug fraction using the Hyperquad simulation and speciation software (HySS, version 4.0.31) [50]. The software determines the amount of free drug by solving mass balance equations using the AAG binding affinities determined in this study (Table 3) and the previously reported HSA–drug *K*_d_ values, which are summarized in Table 4. As far as we know, this is one of the first reports to consider not only the change in glycosylation, but also the change in the concentration of AAG and the influence of HSA on the free drug concentration of the investigated drugs.

The free drug calculations involved peak (*C*_max_) and trough (*C*_min_) plasma drug levels at steady state or therapeutic levels (Table 5), along with 12 µM (low), 22 µM (medium), 31 µM (high), and 69 µM (acute) AAG plasma levels [57]. 

**Figure 4:**
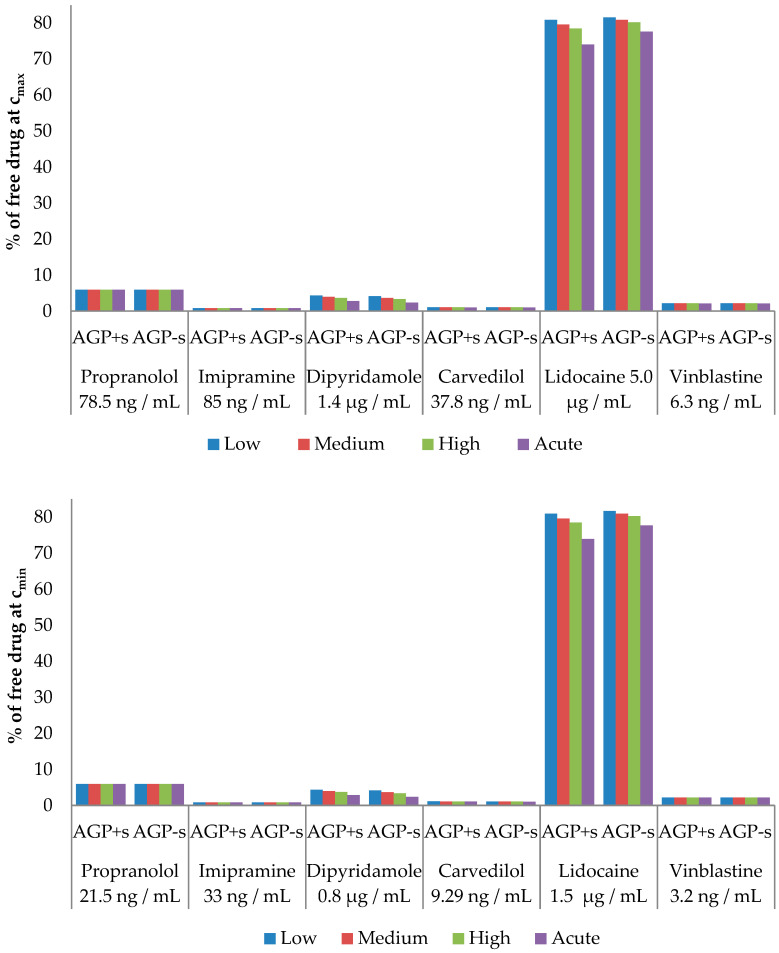
Percentage of free drug at chosen *C*_max_ (**top panel**) and *C*_min_ (**bottom panel**) therapeutic values depending on the plasma concentrations of native AAG + 45 mg/mL HSA (AAG + s) or desialylated AAG + 45 mg/mL HSA (AAG − s).

**Figure 5:**
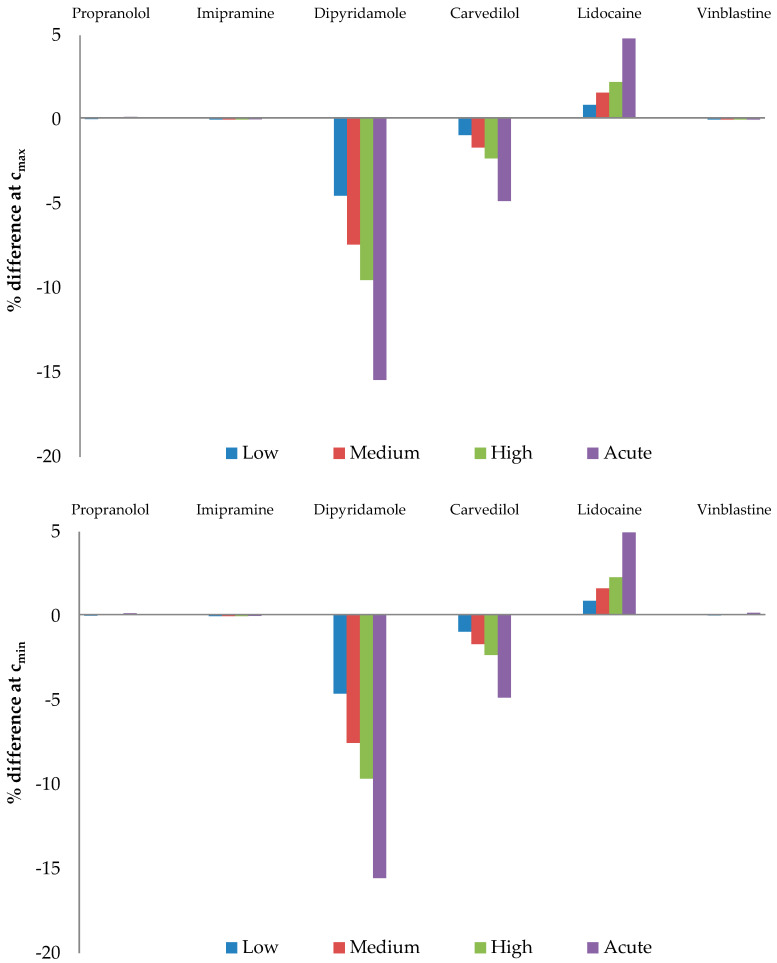
Percentage difference of free drug at *C*_max_ and *C*_min_. Values are calculated versus the native AAG as reference.

The calculated free drug concentrations for both native (AAG + s) and desialylated AAG (AAG − s) were determined based on *C*_max_ and *C*_min_ drug concentrations, as well as AAG concentrations, as depicted in Figure 4. Figure 5 shows the percentage difference in free drug at *C*_max_ and *C*_min_ compared to the native form. Our calculations reveal that desialylation may either increase or decrease free drug concentration in cases where the drug of interest binds to AAG with higher affinity (lower *K*_d_) than to HSA, as seen in dipyridamole, lidocaine, and carvedilol. Conversely, minimal change in free drug for propranolol, imipramine, and vinblastine indicates strong buffering capacity of albumin for these drugs. It is worth noting that, except for lidocaine, all drugs in this study are reported to be highly bound to plasma proteins (>95%), which is consistent with our calculations [60,64,65,66,67,68]. The discrepancy between calculated and experimental lidocaine-free drug levels could be attributed to its interactions with lipoproteins, which were not considered in our calculations.

Only the free drug concentration of dipyridamole may be significantly affected by AAG concentration and/or changes in glycosylation. Dipyridamole is a phosphodiesterase inhibitor that blocks the uptake and metabolism of adenosine by erythrocytes, thrombocytes, and vascular endothelial cells and enhances the antiaggregatory effect of prostacyclin [69]. It is used in the secondary prevention of stroke in combination with acetylsalicylic acid, as an adjunct to warfarin post-mechanical heart valve replacement and in the assessment of coronary artery disease during pharmacological stress testing [70,71]. Therefore, the factors that may influence the free drug concentration of dipyridamole are of great interest due to its immense and long-term use in therapy. Our results show that the free drug concentration of dipyridamole decreases by up to 15%, which may influence the quality of therapy and in certain cases requires correction of the dosage according to the principle of personalized medicine.

The implications of these findings become even clearer when pathological conditions that alter AAG levels are considered. Elevated AAG levels are observed in various clinical scenarios, including inflammation, cancer, and chronic diseases. These conditions can lead to changes in AAG glycosylation patterns, potentially affecting the pharmacokinetics of drugs [5,7,9,10].

Inflammatory diseases such as rheumatoid arthritis or chronic infections often lead to increased AAG levels. This upregulation could increase the binding of drugs such as dipyridamole and carvedilol to AAG, reducing their free concentration and possibly requiring dose adjustment. In addition, many cancers are associated with elevated AAG levels, which may affect the distribution and efficacy of chemotherapeutic agents [13]. Our results suggest that drugs such as vinblastine may have an altered binding profile in cancer patients, potentially impacting therapeutic outcomes. It is also known that chronic diseases such as liver cirrhosis or renal failure can alter AAG concentrations and glycosylation [13]. The effects on drug binding and free drug concentration under such conditions need to be further explored, especially for drugs with high AAG affinity.

## 4. Conclusions

In this study, we used MST to quantitatively evaluate the effects of desialylation on the binding affinity of AAG for different drugs. Our results indicate that desialylation strongly affects the binding affinity of AAG for drugs, resulting in either a decrease or an increase in the *K*_d_ value. These changes are attributed to conformational changes in AAG, a hypothesis supported by thermal shift assays showing increased thermal stability in desialylated AAG. A consistent stoichiometry of 1:1 was observed between AAG and the drugs, indicating a specific interaction. It should be noted that this study utilized a single AAG lot from human plasma sourced in the USA from FDA-approved facilities, introducing potential limitations in clinical applicability due to inherent AAG compositional variability between lots. This variability may arise from differences in glycan structures and aminoacid sequence. The exact impact of such variability on our results cannot be definitively established without further comparative studies. The high sialylation and branching typically observed in AAG from healthy individuals suggest that our sample likely represents this demographic. Investigating AAG variability across diverse demographic groups could provide insights into AAG’s differential behavior under varying biological conditions.

Another aspect yet to be thoroughly investigated is the drug binding affinity to the different genetic variants of AAG, namely ORM1 and ORM2. The effect of altered sialylation or glycosylation on drug interactions with these variants remains largely unexplored. As highlighted earlier, the molar ratio of ORM1 to ORM2 in healthy individuals ranges from about 2 to 3:1, but this can increase significantly in disease states, up to 8:1. Such variations in the molar ratios of AAG genetic variants between lots could also affect experimental outcomes. Therefore, conducting further drug binding studies with isolated AAG variants, considering their different genetic and glycosylation profiles, would be highly valuable. This approach would enhance the understanding of the biological and clinical implications of AAG variability and its impact on drug pharmacokinetics.

The results of the free drug concentration calculations highlight the potential influence of the sialylation status of AAG, particularly in cases where a drug has a greater affinity for AAG than for HSA. This could have significant implications for pharmacokinetics, especially for drugs such as dipyridamole, where altered AAG binding can significantly affect therapeutic efficacy. These results shed light on the intricate interplay of glycosylation patterns, protein conformation, and specificity of drug binding in the complex environment of plasma proteins. The interplay of AAG and HSA in drug binding confirms the complexity of protein–drug interactions in serum.

The Importance of plasma protein binding in pharmacology is an area of great interest. Drugs that are strongly bound and have a low volume of distribution may exhibit pharmacokinetic effects due to variations in protein concentration or other binding-related factors. In this study, we have shown that in some cases, sialylation directly affects the concentration of free drugs. This finding suggests that further pharmacokinetic studies are required to assess the implications of these results, particularly regarding the potential for dose adjustment in personalized medicine for dipyridamole.

Furthermore, the application of MST in this study emphasizes its efficacy as a cost-effective and reliable method for assessing drug–protein interactions. Its utility extends to both existing pharmacologic agents and novel drug candidates, providing a powerful tool for more comprehensive pharmacokinetic analyses. This approach has the potential to significantly advance personalized medicine and contribute to more effective and safer drug therapies tailored to individual patient profiles. Future research should focus on a comprehensive analysis of AAG alterations in different populations, diseases, and their impact on drug efficacy and safety. Such studies would provide valuable insights to optimize drug therapy in the clinical setting to improve the therapeutic index while minimizing adverse effects.

## Figures and Tables

**Table 2:** Molar absorption coefficients of native and desialylated AAG.

AAG	$\frac{\bar{\varepsilon_{28o}}}{M^{-1}{cm}^{-1}}$	*n*	*p*-Value
Native	38,200 ± 340	3	0.97
Desialylated	38,210 ± 60	3	

Uncertainties are reported as the standard deviation; *n* is the number of replicates.

**Table 3:** The equilibrium binding affinities of drugs to native and desialylated AAG.

Drug	*K*_d_/M		*n*	*p*-Value	% Difference
	Native AAG	Desialylated AAG			
Carvedilol	1.17 × 10^−4^ ± 1.40 × 10^−5^	6.35 × 10^−5^ ± 6.75 × 10^−6^	2	0.040 (**)	−45.6
Diltiazem	6.33 × 10^−4^ ± 5.46 × 10^−5^	6.87 × 10^−4^ ± 1.17 × 10^−4^	2	0.617	8.46
Dipyridamole	4.70 × 10^−6^ ± 5.89 × 10^−7^	3.26 × 10^−6^ ± 9.75 × 10^−7^	3	0.094 (*)	−30.6
Imipramine	1.22 × 10^−3^ ± 5.32 × 10^−5^	1.65 × 10^−3^ ± 8.65 × 10^−3^	2	0.027 (**)	35.2
Lidocaine	4.87 × 10^−4^ ± 4.36 × 10^−5^	8.98 × 10^−4^ ± 1.84 × 10^−4^	2	0.091 (*)	84.6
Propranolol	8.11 × 10^−4^ ± 5.69 × 10^−5^	1.20 × 10^−3^ ± 7.81 × 10^−5^	2	0.029 (**)	48.5
Vinblastine	2.22 × 10^−4^ ± 1.50 × 10^−5^	3.08 × 10^−4^ ± 2.98 × 10^−5^	2	0.068 (*)	38.4

Statistical significance of the observed differences is encoded as follows: * *p* < 0.1; ** *p* < 0.05. Uncertainties are reported as standard deviations; *n*—number of replicates. *K*_d_ is calculated by fitting the MST experimental data at pH 7.4 and room temperature.

**Table 4:** HSA–drug *K*_d_ values used for free drug calculation.

Drug	*K*_d_/M
Propranolol ^1^	4.37 × 10^−5^
Imipramine ^2^	6.25 × 10^−6^
Dipyridamole ^3^	3.56 × 10^−5^
Carvedilol ^4^	8.33 × 10^−5^
Lidocaine ^5^	3.23 × 10^−3^
Vinblastine ^6^	1.56 × 10^−5^

^1^ [51], ^2^ [52], ^3^ [53], ^4^ [54], ^5^ [55], ^6^ [56].

**Table 5:** *C*_max_ and *C*_min_ values used for the investigated free drug calculation.

Drug	*C*_max_	*C*_min_
Propranolol ^1^	78.5 ng/mL	21.5 ng/mL
Imipramine ^2^	85.0 ng/mL	33.0 ng/mL
Dipyridamole ^3^	1.40 µg/mL	0.80 µg/mL
Carvedilol ^4^	37.8 ng/mL	9.28 ng/mL
Lidocaine ^5^	1.50 µg/mL	5.00 µg/mL
Vinblastine ^6^	3.20 ng/mL	6.30 ng/mL

^1^ *C*_max_ and *C*_min_ were obtained from six healthy control subjects following oral administration of 80 mg of propranolol twice a day for 7 days [58]. ^2^ *C*_max_ and *C*_min_ are the mean plasma steady state concentrations after 10 days of oral administration of imipramine three times a day [59]. ^3^ *C*_max_ and *C*_min_ are mean peak and trough plasma steady state concentrations following oral administration of 50 mg of dipyridamole every 8 h in six healthy subjects [60]. ^4^ *C*_max_ and *C*_min_ are peak and trough plasma steady state concentrations of both enantiomers following administration of 12.5 mg of carvedilol twice a day for ≥ two weeks in patients with heart failure [61]. ^5^ *C*_max_ and *C*_min_ represent therapeutic drug range [62]. ^6^ *C*_max_ and *C*_min_ represent maximal and minimal plasma concentration after bolus intravenous dose of 3 mg/m^2^ in 16 patients with non-small cell lung cancer [63].

## Data Availability

All data are contained within this manuscript.
